# Strong cis-acting expression quantitative trait loci for the genes encoding SNHG5 and PEX6

**DOI:** 10.1097/MD.0000000000005793

**Published:** 2016-12-30

**Authors:** Jihyeon Lee, Jihye Ryu, Chaeyoung Lee

**Affiliations:** Department of Bioinformatics and Life Science, Soongsil University, 369 Sangdo-ro, Dongjak-gu, Seoul, Korea.

**Keywords:** chromosome 6, eQTL, gene expression, PEX6, SNHG5, SNP

## Abstract

Expression of quantitative trait loci (eQTLs) for the genes located in human chromosome 6 were examined. Data on RNA expression in lymphoblastoid cells of 373 unrelated Europeans were used to identify eQTLs.

Genome-wide analysis resulted in 24,447 nucleotide variants associated with gene expression (*P* < 2.16 × 10^−10^). We found 36variants with *P* < 10^−100^, which were all associated with expression levels of the genes encoding small nucleolar RNA host gene 5 (SNHG5) and peroxisomal biogenesis factor 6 (PEX6). Enhancer eQTLs downstream of theSNHG5 gene might be candidate genetic factors for susceptibility to cancer. This is because nucleotide substitutions (eg, G→T at rs6922) of the enhancer eQTLs may cause low expression of SNHG5 gene, and low expression of snoRNA U50, a product generated from introns of the SNHG5gene, can induce cancer. One presently identified eQTL for the PEX6 gene was rs10948059, which had been associated with prostate cancer from previous association studies. The results imply that variants associated with prostate cancer can be identified through expressional change in the PEX6 gene, but not in the overlapped glycine N-methyltransferase gene which had been considered as a candidate gene.

Further studies are required to understand their underlying mechanisms for the strong eQTLs for the SNHG5 and PEX6 genes.

## Abstract 2

Supplemental Digital Content is available in the text

## 1 Introduction

As of 2003, more than 170 million base pairs had been completely sequenced for human chromosome 6.^[1]^ Genome-wide association studies (GWAS) have unraveled a large number of single-nucleotide variants associated with human traits. According to the GWAS Catalog, 32,768 genetic associations were reported across hundreds of diseases and quantitative traits as of October 2016 (*P* < 10^−5^; https://www.ebi.ac.uk/gwas). The GWAS signals were more frequently found in chromosome 6 than in any other chromosomes, even in chromosome 1, which is 1.5 times longer than chromosome 6. The chromosome 6 has medical significance, with about 120 causal genes reported for complex diseases, such as cancers, heart disease, diabetes, Alzheimer disease, rheumatoid arthritis, and multiple sclerosis.^[2–4]^ In particular, major histocompatibility complex and related regions including the human leukocyte antigen (HLA) genes in chromosome 6 are critically related to infection, immunity, and inflammation.^[2]^ Many nucleotide variants turned out to be important for susceptibility to complex diseases from GWAS. One example is rs9378815, an intergenic single-nucleotide polymorphism (SNP) on chromosome 6, which has been identified as a rheumatoid arthritis risk locus from a trans-ethnic GWAS meta-analysis using >100,000 subjects (*P* = 1.7 × 10^−10^).^[5]^ Another example is rs2046210, an SNP upstream of the gene encoding estrogen receptor alpha on chromosome 6, which was associated with susceptibility to breast cancer in a study involving over 31,000 subjects (eg, *P* = 1.5 × 10^−30^ in Chinese women).^[6]^ Nevertheless, their underlying mechanisms are unknown. Identifying nucleotide variants that influence RNA transcript expression levels is critical to understand underlying mechanisms of GWAS signals. The objective of the current study was to identify expression quantitative trait loci (eQTLs) for genes located in chromosome 6, which might have abundant potential regulatory sequences.

## 2 Methods

The study used RNA expression data for lymphoblastoid cells of 376 unrelated individuals from European populations of CEPH (CEU), Finns (FIN), British (GBR), and Toscani (TSI) to identify regulatory variants for expression of the 573 genes in chromosome 6, which were obtained from the study by Lappalainen et al.^[7]^ The genes included protein-coding genes and long intergenic noncoding RNA genes. The expression level was calculated as the sum of reads per kilobase of transcript per million mapped reads values of all the transcripts of each gene for each individual. A read was counted in an exon if either start or end coordinate of the read was in the exon. To avoid overestimating gene expressions, the count was divided by the number of overlapping exons among split reads. Their corresponding genotypes were obtained from the 1000 Genomes project (phase I; http://phase1browser.1000genomes.org/) that originally conducted whole genome sequencing with an average depth of 5×, and whole exome sequencing with an average depth of 80× using 1092 individuals from 14 populations of Europeans, East Asians, Saharan Africans, and Americans. A total of 404,240 SNPs on chromosome 6 among 5,941,815 genome-wide SNPs were included in the current analysis after a filtration process (excluding SNPs with minor allele frequency [MAF] <0.05). Three individuals with missing rate >0.05 of the genotypes were excluded from the analysis. Ethical approval was not necessary because we dealt with publically available data.

Genetic associations of SNPs with RNA expression were conducted to determine the significance of regression coefficient for minor allele effect. We employed the following mixed model incorporating a genomic relationship matrix (GRM) that reflected polygenic covariance among individuals to avoid population stratification^[8]^:
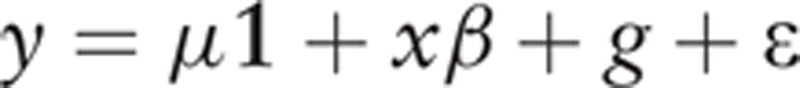


where *y* is the vector of gene expression levels, *μ* is the overall mean, 1 is the vector of 1 s, *β* is the fixed effect for minor allele of the SNP to be tested for association, and *x* is the vector of the numbers of minor alleles of the SNP, for example, 0(0, 0) for homozygote of the major allele, 1 (1, 0) for heterozygote, and 2 (1, 1)for homozygote of the minor allele in additive (dominant, recessive) model. Vector *g* is the vector of random polygenic effects with 
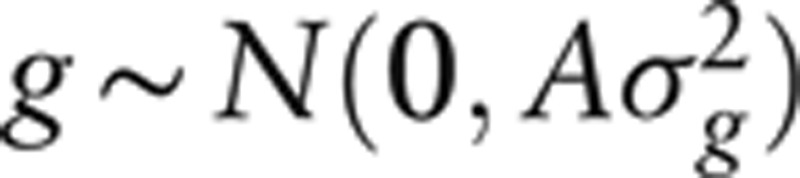
, where *A* is GRM estimated using SNPs, and 
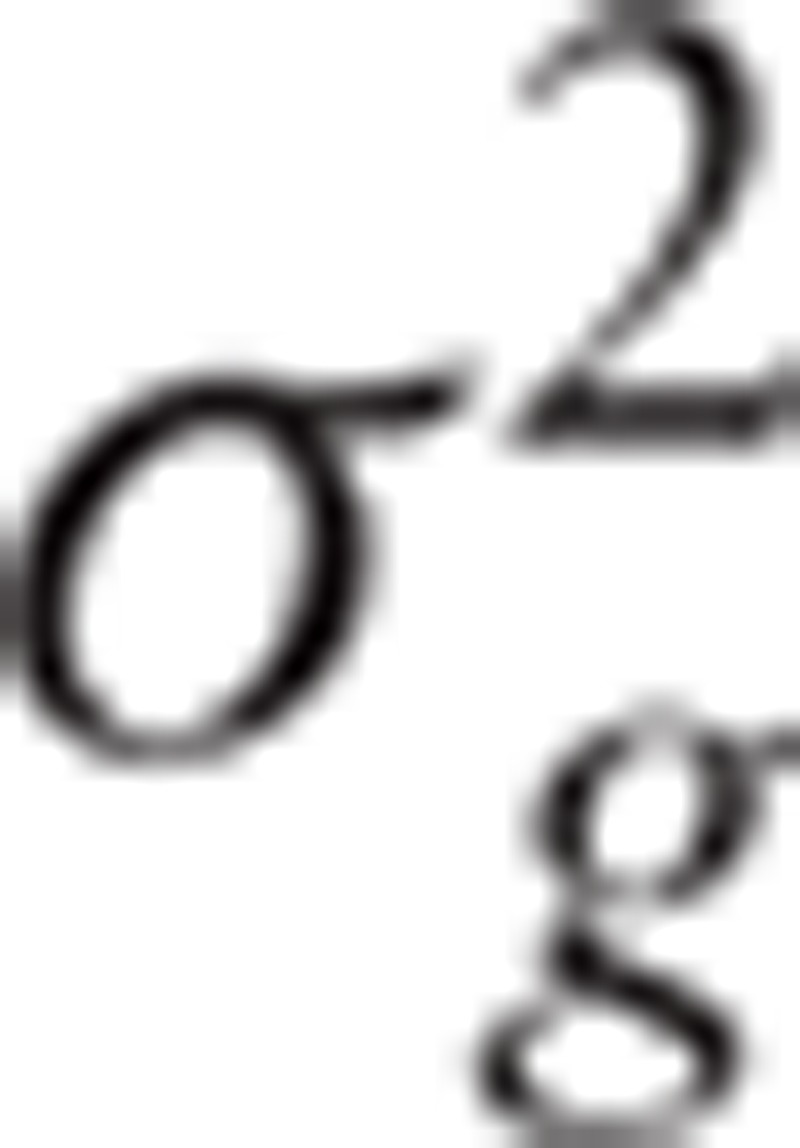
 is the polygenic variance component. Elements of *A* consist of the following pair-wise genomic relationship coefficients estimated using genotypes of SNPs in linkage equilibrium (*r*^2^ > 0.8): 
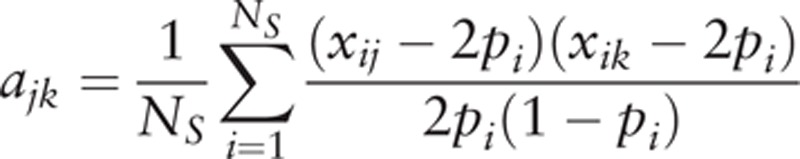


where *a*_*jk*_ is the genomic relationship coefficient between *j*^th^ and *k*^th^ individuals, *N*_*S*_ is the number of SNPs, *x*_*ij*_ (*x*_*ik*_) is the number (0, 1, or 2) of the minor allele at the *i*^th^ SNP for the *j*^th^ (*k*^th^) individual, *p*_*i*_ is the frequency of the minor allele at the *i*^th^ SNP. *ε* is the vector of random residuals with 
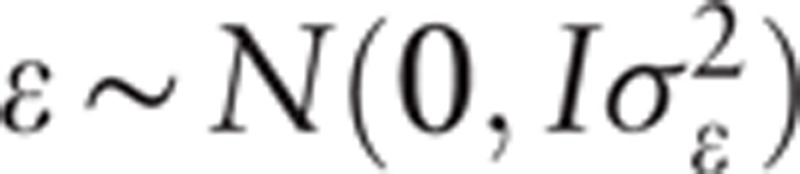
, where 
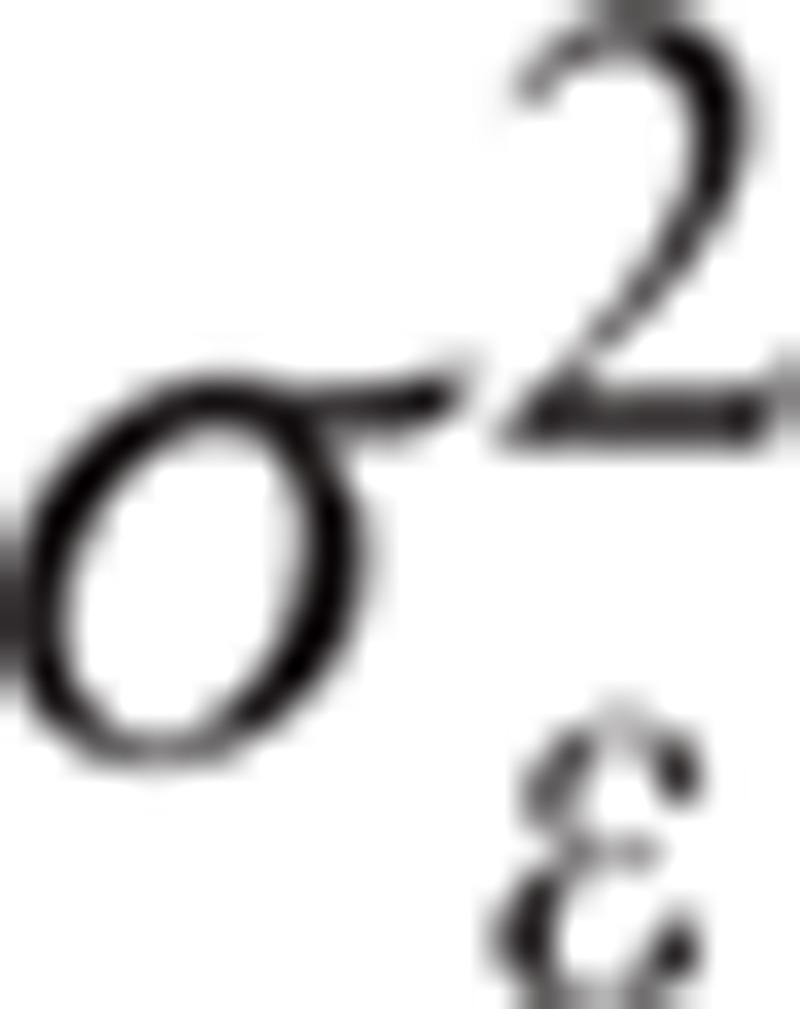
 is the residual variance component and *I* is the identity matrix. All the association analyses were conducted using Genome-wide Complex Trait Analysis v1.24 freeware.^[9]^

Multiple testing was applied employing Bonferroni correction. A total of 231,629,520 association tests for 573 genes and 404,240 SNP markers resulted in the significance threshold value of 2.16 × 10^−10^. The association tests were analyzed using PLINK ver.1.04 (Free Software Foundation, Inc. Boston, MA).

Linkage disequilibrium (LD) blocks were constructed based on the algorithm of Gabriel et al^[10]^ (Haploview version 4.2). The algorithm used a 95% confidence interval (CI) of pair-wise D’ estimate between SNPs with MAF >0.05.

Promoters/enhancers including the significant SNPs were examined using genome-wide chromatin interaction analysis with paired-end-tag sequencing (ChIA-PET) data, which resulted from the study by Li et al.^[11]^ The paired-end-tag (PET) sequences were mapped with the human reference genome of hg19. High-confidence intrachromosomal PET clusters with *P*_FDR_ <0.05were included with genomic span of 8 Kb to 1 Mb and PET count >3 for each PET cluster from the saturated libraries. Interaction anchors flanking transcription start site ±5 Kb were defined as promoters.

Enrichment analysis was conducted to analyze associations of significant SNPs with diseases and biological processes using genomic regions enrichment of annotations tool.^[12]^ Diseases and biological processes based on the Gene Ontology (http://www.geneontology.ogr/) were used as input annotation terms. Each gene involved in input annotation terms was assigned to a regulatory domain that extends up to 1 Mb in both directions. Significance of enrichment was determined by a hypergeometric test over genes. The significance threshold was adjusted by false discovery rate (FDR) to control false-positives.

Other enrichment analyses were also performed to determine enrichment of cis versus trans regulation, promoter/enhancer versus nonpromoter/nonenhancer regions, and downstream versus upstream regions. Their significances were all determined with *P* < 0.01 by the Fisher exact test.

## 3 Results

**Figure 1:**
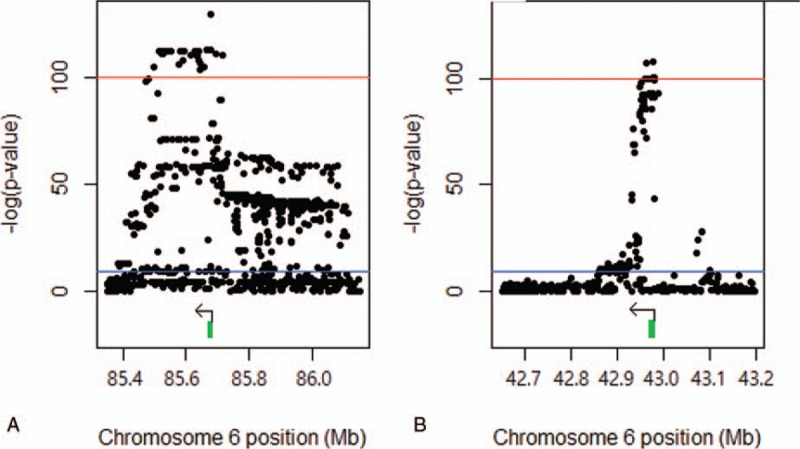
Genetic associations of nucleotide variants with mRNA expression of SNHG5 (A) and PEX6 (B).The blue line indicates the significance threshold with *P* < 2.16 × 10^−10^, and the red line indicates the significance threshold with *P* < 10^−100^. The green bar and the black arrow indicate region and direction of each gene, respectively.

**Figure 2:**
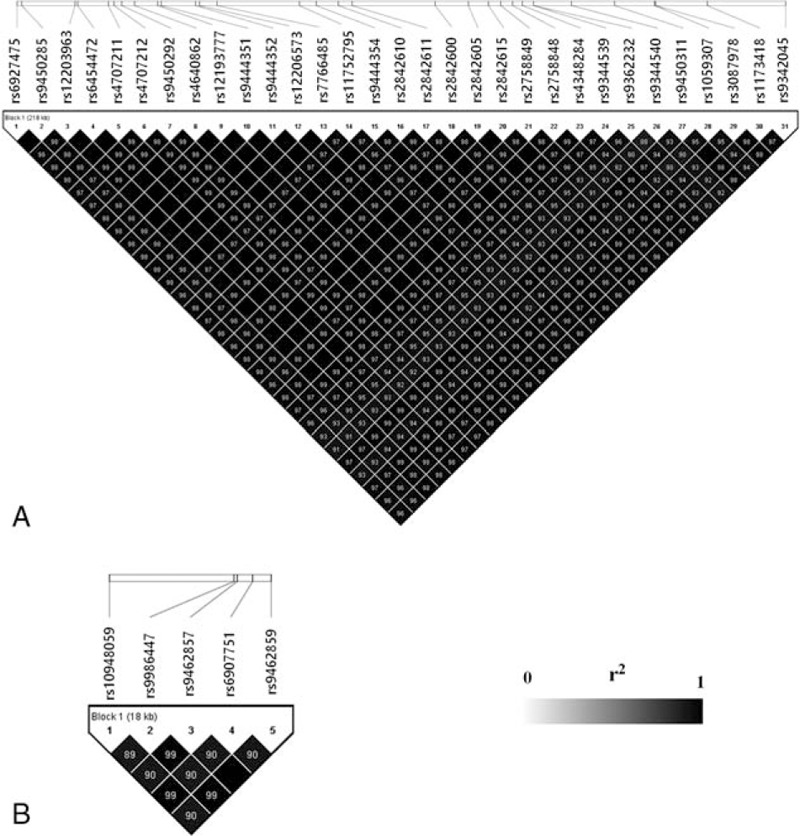
Linkage disequilibrium blocks for strongly significant SNPs (*P* < 10^−100^) in and around the SNHG5 (A) and PEX6 (B) genes.

The eQTL analysis revealed that 24,447 SNPs were associated with expression of the 88 genes located on chromosome 6 (*P* < 2.16 × 10^−10^). Most were identified using the additive model (24,213 SNPs). They were enriched (*P*_FDR_ < 0.05, where *P*_FDR_ is *P* value adjusted by FDR) with genes involved in a variety of diseases (Supplementary Table 1) and biological processes (Supplementary Table 2).The SNPs were enriched (*P* < 0.01) in promoter and enhancer regions resulting from a ChIA-PET study.^[11]^ There were 18,872 cis-eQTLs within 1 Mb from target genes and 5575trans-eQTLs >1 Mb away from target genes, and they were largely cis-regulatory eQTLs (77% = 18,872/24,447) by the enrichment analysis (*P* < 0.01). The cis-eQTLs appeared considerably clustered with 68% (12,886/18,872) located within 100 Kb. Also, 4960 SNPs were associated with expression of multiple genes, which resulted in 18,378 unique SNPs. In particular, 36 SNPs were observed with extremely low *P* value (*P* < 10^−100^; Table 1). All 36 SNPs influenced RNA expression of the genes encoding small nucleolar RNA host gene 5 (SNHG5) and peroxisomal biogenesis factor 6 (PEX6). The SNPs were all located in or near the corresponding gene. On chromosome 6, the SNHG5 gene is located from 85,676,990 to 85,678,736 bp (GRCh38/hg38), and the PEX6 gene is located from 42,964,335 to 42,979,150 bp (GRCh38/hg38). Considerable numbers of SNPs flanking the genes were associated with their expression (*P* < 2.16 × 10^−10^; Fig. 1). Most of the strongly significant SNPs with *P* < 10^−100^ were found downstream of the SNHG5 gene and within the PEX6 gene. The SNPs were observed in strong linkage (*r*^2^ > 0.8), forming LD blocks (Fig. 2).

**Table 1:**
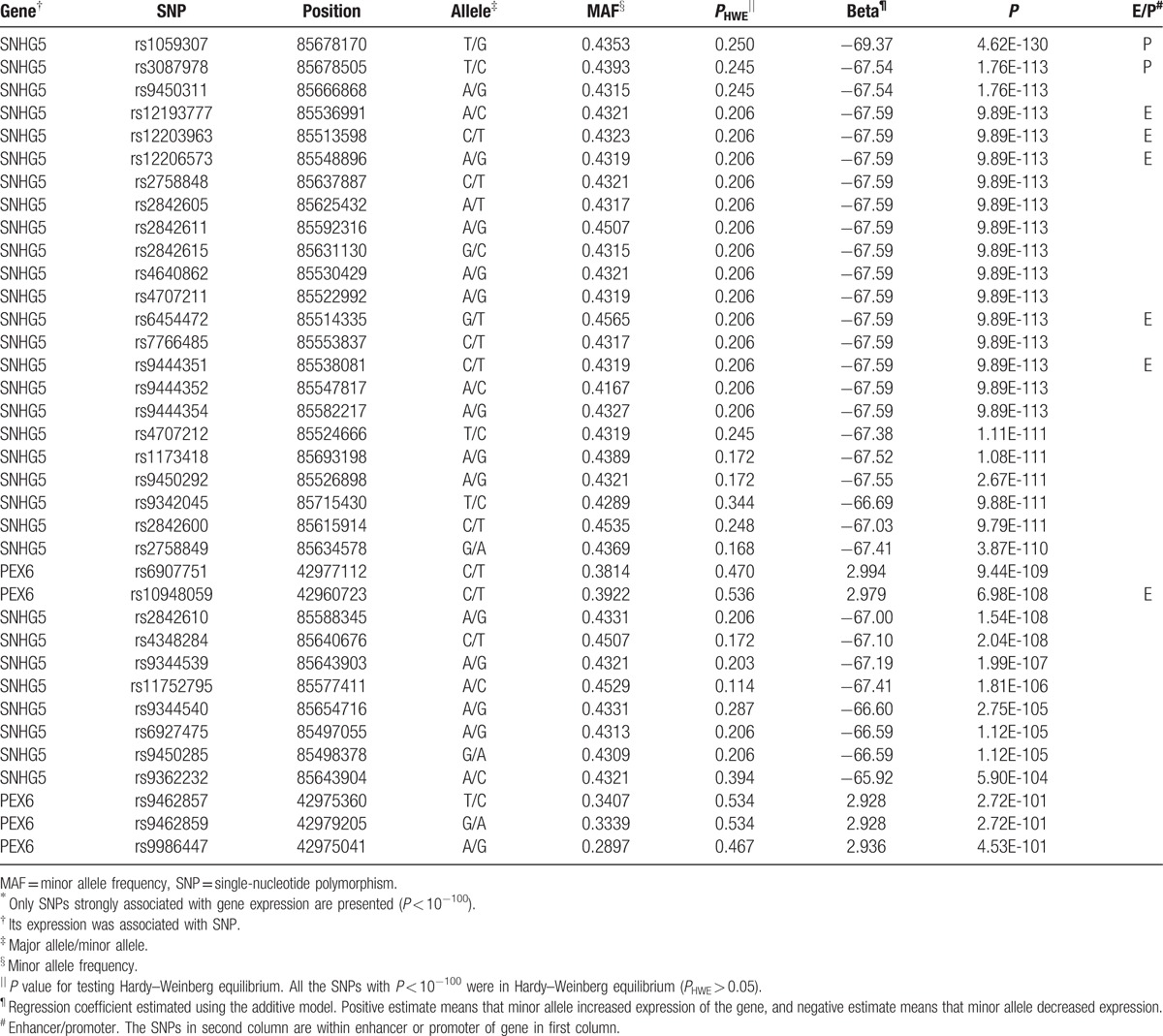
Genetic associations of nucleotide variants with mRNA expression of genes located in chromosome 6^∗^.

## 4 Discussion

The current study identified 24,447 SNPs associated with RNA expression of genes located in chromosome 6, including cis-eQTLs (77%) that may affect transcription initiation complex on the same physical chromosome. The cis-eQTLs tended to be considerably clustered with 68% located within 100 Kb from their corresponding target gene, which concurred with the previous study where 80% of cis-eQTLs was identified within 100 Kb using 9 types of tissues.^[13]^

The current study showed the pleiotropy that 4960 SNPs were associated with expression of multiple genes. This implied direct or indirect functional relationships among genes simultaneously regulated by eQTLs. For example, rs2020202 was associated with expression of 9 histone genes (HIST1B, HIST1E, HIST2AH, HIST2AJ, HIST2BM, HIST3B, HIST3I, HIST3J, and HIST4B), 26 SNPs were associated with expression of 3 HLA genes (HLA-DQB2, HLA-DRB6, and HLA-DRB1), and 9 SNPs were associated with expression of 3 serpin genes (SERPINB1, SERPINB6, and SERPINB9).

In particular, 36 SNPs showed strong associations with *P* < 10^−100^, and they were all associated with expression of the gene encoding SNHG5 or PEX6. All the SNPs were located flanking the corresponding gene within 200 Kb. An enrichment analysis showed that the SNPs strongly associated with expression of the SNHG5 were enriched downstream of the gene (*P* < 0.01). However, SNPs with *P* < 2.16 × 10^−10^ were enriched upstream of the gene (*P* < 0.01).The difference in the degree of significance implied that downstream and upstream signals may act as regulatory sequences with different importance. The association signal of rs6922 (*P* = 1.26 × 10^−54^) was located far from the target gene (SNHG5; 181,402 bp apart), and thus it was suspected as an enhancer variant. This speculation led us to compare it with the sequences resulting from a ChIA-PET study.^[11]^ As a result, we found that 1 anchor of an interacting PET cluster included rs6922,and the other anchor was the promoter of the SNHG5gene. The current studyalso showed that nucleotide substitution from G to T at rs6922 decreased the expression of SNHG5 (beta = −53.7). The SNPs with *P* < 2.16 × 10^−10^ were enriched in the promoter and enhancer regions of the SNHG5 gene (*P* < 0.01; 7 SNPs in the promoter and 23 SNPs in the enhancer regions).

Nucleotide substitution of such variants might influence the expression of SNHG5 gene and is candidate genetic factor for susceptibility to cancer. This is supported by previous studies in which a variety of cancers were induced by low expression of snoRNAU50, a product generated from introns 4 and 5 of the SNHG5 gene. Expression of U50 is down-regulated in prostate and breast cancer samples.^[14,15]^ These studies also showed that the expression of U50 can inhibit colony formation of prostate and breast cancer cells. SNHG5 gene is located at the chromosomal translocation breakpoint involved in B-cell lymphoma (provided by RefSeq, July 2008). It was recently reported that the serum levels of SNHG5 were up-regulated in patients with malignant melanoma, and that SNHG5 may function in melanomagenesis or melanoma metastasis.^[16]^ Further studies are needed to understand the underlying mechanisms of nucleotide substitutions on cancers and their subtypes.

The current study revealed many intragenic eQTLs for the PEX6 gene. The gene encodes a member of the ATPases associated with diverse cellular activities. In particular, the PEX6 plays a key role in biogenesis of peroxisome, which is a small eukaryotic organelle specialized to perform oxidative reactions. Its mutations cause peroxisome biogenesis disorders of complementation groups 4 and 6.^[17]^ This suggests that the eQTLs detected from the current study might influence on susceptibility to the peroxisome biogenesis disorders by abnormal expression of PEX6.

Two of the eQTLs for PEX6 had been identified for association with prostate cancer from 2 previous studies which revealed rs10948059 and rs9462856 linked to it in European populations, respectively.^[18,19]^ These findings were interpreted that the genetic associations with prostate cancer were produced by regulating the expression of the glycine N-methyltransferase (GNMT) gene that overlaps with the PEX6 gene. The authors suspected the GNMT gene because the rs10948059 and rs9462856 were their intronic variants. Since the nucleotide variants were revealed as a strong eQTL for PEX6 gene from the current study (*P* = 6.98 × 10^−108^), the variants associated with prostate cancer might be identified through expressional change in PEX6. This was supported by the results of the Human Protein Atlas in which PEX6 is down-regulated in prostate cancer cells (http://www.proteinatlas.org/ENSG00000124587-PEX6/cancer).

We found that the strong eQTLs (*P* < 10^−100^) using additive model (“AA” vs “AB” vs “BB,” where A is major allele and B is minor allele^[20]^) were all significant using dominance (“AA + AB” vs “BB”) and recessive (“AA” vs “AB + BB”) models (*P* < 2.16 × 10^−10^). However, they were not as strong as observed using the additive model, and all showed *P* > 10^−100^. The best fit into the additive model implied that the expressional regulation of eQTLs was correlated with the number of minor alleles.

The current study also identified eQTLs with 10^−100^ < *P* < 2.16 × 10^−10^. In particular, some eQTLs for PEX6 (rs9471950, rs9471951, rs7754294, rs13203402, rs7740252, rs7758576, and rs7759112; 5.63 × 10^−12^ < *P* < 1.91 × 10^−10^) turned out to be enhancer variants of the serum response factor (SRF) gene, which resulted from chromatin interaction analysis with paired-end-tag using RNA polymerase II.^[11]^ The regulatory variants could be plausible because SRF is a well-known transcript factor for the PEX6 gene.

In this study, we employed a mixed model incorporating pair-wise polygenic relationship among individuals to control population stratification, which may reduce false-positive eQTLs.^[8]^ The analysis revealed strong cis-acting eQTLs for SNHG5 and PEX6, and offered insights about their cellular mechanism of transcriptome variation. However, this study was limited to gene expression in lymphoblastoid cells. Other strong eQTLs would be identified with different cell types. In addition, this study had another limitation in understanding specific functions of eQTLs in gene expression (eg, splicing regulation and transcript stability).

In conclusion, the current study revealed novel eQTLs for SNHG5 and PEX6 genes in chromosome 6.Nucleotide substitutions of the eQTLs might be candidate factors for a variety of cancers by regulating expression of the 2 genes. Further studies are needed to understand their underlying mechanisms.

## Supplementary Material

Supplemental Digital Content
