# Supplementary material for: Strong cis-acting expression quantitative trait loci for the genes encoding SNHG5 and PEX6
Source: Medicine (Baltimore). 2016 Dec 30;95(52):e5793. doi: 10.1097/MD.0000000000005793 (PMC5207599; doi:10.1097/MD.0000000000005793)
Supplement: Supplemental Digital Content [file medi-95-e5793-s001.docx]

**Supplementary table 1.** Enrichment analysis of eQTLs with genes involved in disease^a^

| **Disease** | **P_FDR_**^b^ | **Fold enrichment** |
| --- | --- | --- |
| Psoriasis | 5.22E-08 | 6.27 |
| Skin disease | 9.30E-06 | 3.12 |
| Integumentary system disease | 9.34E-06 | 3.11 |
| Mucocutaneous lymph node syndrome | 1.14E-05 | 10.97 |
| Lymphadenitis | 1.84E-05 | 10.23 |
| Vascular skin disease | 2.07E-05 | 5.76 |
| Graves' disease | 1.07E-04 | 8.05 |
| Thyrotoxicosis | 1.10E-04 | 7.14 |
| Hyperthyroidism | 1.12E-04 | 7.21 |
| Polyarteritis nodosa | 1.24E-04 | 7.84 |
| Goiter | 1.39E-04 | 6.70 |
| Peripheral vascular disease | 1.39E-04 | 4.41 |
| Autoimmune disease | 1.48E-04 | 2.52 |
| Multiple sclerosis | 3.80E-04 | 3.67 |
| Demyelinating disease of central nervous system | 4.49E-04 | 3.61 |
| Allergy | 5.66E-04 | 4.40 |
| Demyelinating disease | 5.91E-04 | 3.49 |
| Cystic echinococcosis | 5.93E-04 | 60.34 |
| Autoimmune disease of endocrine system | 9.93E-04 | 5.80 |
| Intrahepatic cholestasis | 1.02E-03 | 16.76 |
| Lymphatic system disease | 1.56E-03 | 2.16 |
| Cavernous hemangioma | 1.81E-03 | 45.25 |
| Congenital adrenal hyperplasia | 2.16E-03 | 21.94 |
| Behcet's disease | 2.55E-03 | 6.61 |
| Neuromyelitis optica | 7.81E-03 | 16.09 |
| Liver disease | 1.12E-02 | 2.30 |
| Prostatitis | 1.16E-02 | 25.86 |
| Vascular disease | 1.38E-02 | 1.86 |
| Urticaria | 1.39E-02 | 6.96 |
| Hypotrichosis | 1.39E-02 | 6.96 |
| Immunoproliferative disease | 1.40E-02 | 2.00 |
| Hepatitis | 1.41E-02 | 2.59 |
| Cytomegalovirus infectious disease | 1.43E-02 | 4.45 |
| Lymphoproliferative disease | 1.47E-02 | 2.00 |
| Glucose metabolism disease | 1.47E-02 | 1.99 |
| Echinococcosis | 1.47E-02 | 22.63 |
| Alcoholic pancreatitis | 1.47E-02 | 22.63 |
| Pure red-cell aplasia | 1.49E-02 | 60.34 |
| Pemphigus | 1.50E-02 | 8.62 |
| Psoriatic arthritis | 1.50E-02 | 8.62 |
| Adrenal hyperplasia | 1.51E-02 | 12.07 |
| Hair disease | 1.52E-02 | 6.46 |
| Flaviviridae infectious disease | 1.52E-02 | 3.13 |
| Diabetes mellitus | 1.53E-02 | 2.00 |
| Sarcoidosis | 1.54E-02 | 5.41 |
| Bone inflammation disease | 1.56E-02 | 2.17 |
| Dengue shock syndrome | 1.60E-02 | 20.11 |
| Pancreas disease | 1.65E-02 | 1.95 |
| Arthritis | 1.67E-02 | 2.19 |
| Nasopharynx carcinoma | 1.68E-02 | 3.42 |
| Lymphoid cancer | 1.69E-02 | 1.97 |
| Leprosy | 1.70E-02 | 7.94 |
| Endocrine pancreas disease | 1.78E-02 | 1.96 |
| Vasculitis | 1.88E-02 | 3.96 |
| Hepatitis C | 2.02E-02 | 3.12 |
| Carbohydrate metabolism disease | 2.13E-02 | 1.90 |
| Brucellosis | 2.14E-02 | 7.39 |
| Leukemia | 2.31E-02 | 1.85 |
| Reactive arthritis | 2.32E-02 | 16.46 |
| Arthropathy | 2.37E-02 | 4.80 |
| Ankylosing spondylitis | 2.38E-02 | 5.66 |
| Spondylitis | 2.38E-02 | 5.66 |
| Vitiligo | 2.38E-02 | 5.66 |
| Malignant neoplasm of lymphatic and hemopoietic tissue | 2.72E-02 | 1.74 |
| Bacterial prostatitis | 2.75E-02 | 40.23 |
| Aplastic anemia | 2.77E-02 | 5.40 |
| Alopecia | 2.78E-02 | 6.70 |
| Endocrine system disease | 2.98E-02 | 1.71 |
| Pulmonary tuberculosis | 2.99E-02 | 6.56 |
| Purpura | 3.06E-02 | 5.25 |
| Schizophrenia | 3.09E-02 | 2.08 |
| Psychotic disease | 3.17E-02 | 2.07 |
| Chronic leukemia | 3.20E-02 | 2.61 |
| Bone disease | 3.25E-02 | 1.92 |
| Uveitis | 3.25E-02 | 8.62 |
| Cardiovascular system disease | 3.33E-02 | 1.47 |
| Primary bacterial infectious disease | 3.43E-02 | 2.83 |
| (+)ssRNA virus infectious disease | 3.54E-02 | 2.68 |
| Hematologic cancer | 3.56E-02 | 1.65 |
| Spondyloarthropathy | 3.61E-02 | 4.96 |
| Dermatitis | 3.85E-02 | 2.64 |
| Hepatitis B | 3.89E-02 | 3.13 |
| Hepatobiliary disease | 4.14E-02 | 1.94 |
| Alloimmunization | 4.18E-02 | 30.17 |
| Vascular hemostatic disease | 4.18E-02 | 2.73 |

^a^Enrichment was determined by hypergeometric test. Enrichment was analyzed using GREAT v3.0.0 (<http://bejerano.stanford.edu/great/public/html>). Diseases only with P_FDR_ < 0.05 are presented.

^b^P-value adjusted for multiple testing by false discovery rate

**Supplementary table 2.** Enrichment analysis of eQTLs with genes involved in biological process^a^

| **Biological process** | **P_FDR_**^b^ | **Fold enrichment** |
| --- | --- | --- |
| Nucleosome assembly | 5.04E-27 | 14.67 |
| Chromatin assembly | 5.09E-26 | 13.54 |
| Nucleosome organization | 3.16E-25 | 12.65 |
| Protein-DNA complex assembly | 3.38E-25 | 12.72 |
| Chromatin assembly or disassembly | 1.40E-24 | 12.07 |
| DNA packaging | 3.41E-23 | 10.94 |
| Antigen processing and presentation | 9.64E-13 | 7.58 |
| Antigen processing and presentation of peptide antigen | 3.28E-11 | 7.67 |
| Positive regulation of T cell mediated cytotoxicity | 1.07E-10 | 25.53 |
| Positive regulation of leukocyte mediated cytotoxicity | 6.74E-10 | 18.57 |
| Regulation of T cell mediated cytotoxicity | 9.47E-10 | 21.41 |
| Positive regulation of cell killing | 1.60E-09 | 17.24 |
| Antigen processing and presentation of exogenous peptide antigen | 2.27E-09 | 7.36 |
| Cellular response to interferon-gamma | 2.94E-09 | 11.04 |
| Response to interferon-gamma | 4.36E-09 | 9.65 |
| Antigen processing and presentation of exogenous antigen | 4.44E-09 | 7.06 |
| Regulation of leukocyte mediated cytotoxicity | 5.33E-09 | 15.41 |
| Interferon-gamma-mediated signaling pathway | 8.02E-09 | 12.86 |
| Protein complex assembly | 8.11E-09 | 3.12 |
| Protein complex biogenesis | 8.34E-09 | 3.11 |
| Positive regulation of T cell mediated immunity | 9.61E-09 | 17.02 |
| Regulation of cell killing | 9.96E-09 | 14.48 |
| Antigen processing and presentation of peptide or polysaccharide antigen via MHC class II | 2.43E-08 | 9.33 |
| Positive regulation of immune response | 3.22E-08 | 4.17 |
| Macromolecular complex assembly | 3.27E-08 | 2.83 |
| Positive regulation of lymphocyte mediated immunity | 6.28E-08 | 12.27 |
| Positive regulation of leukocyte mediated immunity | 9.20E-08 | 11.87 |
| Regulation of T cell mediated immunity | 1.75E-07 | 13.01 |
| Protein complex subunit organization | 2.99E-07 | 2.60 |
| Positive regulation of immune system process | 6.41E-07 | 3.27 |
| Regulation of immune system process | 6.53E-07 | 2.60 |
| Positive regulation of adaptive immune response based on somatic recombination of immune receptors built from immunoglobulin superfamily domains | 6.75E-07 | 11.44 |
| Macromolecular complex subunit organization | 1.05E-06 | 2.41 |
| Positive regulation of adaptive immune response | 1.13E-06 | 10.88 |
| Cellular component assembly | 1.30E-06 | 2.27 |
| Antigen processing and presentation of peptide antigen via MHC class I | 2.67E-06 | 7.84 |
| Immune response | 5.54E-06 | 2.45 |
| Regulation of immune effector process | 9.99E-06 | 4.57 |
| Cellular component biogenesis | 1.10E-05 | 2.11 |
| Regulation of lymphocyte mediated immunity | 1.11E-05 | 7.70 |
| Positive regulation of immune effector process | 1.31E-05 | 6.82 |
| Antigen processing and presentation of exogenous peptide antigen via MHC class I, TAP-independent | 1.41E-05 | 37.71 |
| Negative regulation of megakaryocyte differentiation | 4.46E-05 | 21.30 |
| Antigen processing and presentation of exogenous peptide antigen via MHC class II | 5.22E-05 | 7.46 |
| Antigen processing and presentation of peptide antigen via MHC class II | 6.44E-05 | 7.29 |
| Regulation of adaptive immune response | 6.98E-05 | 6.46 |
| Regulation of leukocyte mediated immunity | 1.08E-04 | 6.19 |
| Regulation of gene silencing | 1.14E-04 | 13.62 |
| Regulation of immune response | 1.20E-04 | 2.68 |
| Immune system process | 1.80E-04 | 1.92 |
| Regulation of adaptive immune response based on somatic recombination of immune receptors built from immunoglobulin superfamily domains | 1.97E-04 | 6.44 |
| Innate immune response | 2.95E-04 | 2.56 |
| Antigen processing and presentation of exogenous peptide antigen via MHC class I, TAP-dependent | 5.69E-04 | 7.34 |
| Antigen processing and presentation of exogenous peptide antigen via MHC class I | 8.36E-04 | 6.96 |
| T cell costimulation | 1.03E-03 | 7.91 |
| Lymphocyte costimulation | 1.15E-03 | 7.79 |
| T cell receptor signaling pathway | 1.31E-03 | 6.54 |
| Activation of immune response | 1.88E-03 | 3.23 |
| Positive regulation of T cell activation | 2.53E-03 | 4.41 |
| Regulation of T cell activation | 4.05E-03 | 3.67 |
| Lymphocyte mediated immunity | 5.41E-03 | 5.43 |
| Leukocyte mediated immunity | 6.41E-03 | 4.75 |
| Positive regulation of response to stimulus | 7.52E-03 | 1.91 |
| Defense response | 9.04E-03 | 1.91 |
| Antigen receptor-mediated signaling pathway | 1.17E-02 | 4.89 |
| T cell mediated immunity | 1.78E-02 | 14.20 |
| Interferon-gamma secretion | 1.78E-02 | 25.86 |
| Immune response-activating cell surface receptor signaling pathway | 1.86E-02 | 3.81 |
| Positive regulation of lymphocyte activation | 2.72E-02 | 3.40 |
| Cytosol to ER transport | 3.11E-02 | 60.34 |
| Cytokine-mediated signaling pathway | 3.47E-02 | 2.82 |
| Immune response-activating signal transduction | 3.52E-02 | 2.94 |

^a^Enrichment was determined by hypergeometric test. Enrichment was analyzed using GREAT v3.0.0 (<http://bejerano.stanford.edu/great/public/html>). Biological processes only with P_FDR_ < 0.05 are presented.

^b^P-value adjusted for multiple testing by false discovery rate
